# Impact of preoperative skeletal muscle mass and physical performance on short‐term and long‐term postoperative outcomes in patients with esophageal cancer after esophagectomy

**DOI:** 10.1002/ags3.12560

**Published:** 2022-03-08

**Authors:** Keijiro Sugimura, Hiroshi Miyata, Takashi Kanemura, Tomohira Takeoka, Naoki Shinnno, Kazuyoshi Yamamoto, Takeshi Omori, Masaaki Motoori, Masayuki Ohue, Masahiko Yano

**Affiliations:** ^1^ 53312 Department of Digestive Surgery Osaka International Cancer Institute Osaka Japan; ^2^ Department of Surgery Kansai Rosai Hospital Hyogo Japan; ^3^ Department of Surgery Osaka General Medical Center Osaka Japan; ^4^ Department of Surgery Osaka Suita Municipal Hospital Osaka Japan

**Keywords:** 6‐minute walk distance, esophageal cancer, sarcopenia, skeletal muscle index

## Abstract

**Background:**

In patients with esophageal cancer who undergo esophagectomy, preoperative skeletal muscle mass loss has been reported to be associated with postoperative complications and poor prognosis. However, physical performance has not been fully investigated.

**Methods:**

This study included 363 patients who underwent esophagectomy for thoracic esophageal cancer in 2013‐2018. Preoperative skeletal muscle index (SMI) was measured with multifrequency bioelectrical impedance. Preoperative 6‐minute walk distance (6MWD) was measured as an indicator of physical performance. We investigated the association between these factors and postoperative complications or long‐term prognosis.

**Results:**

Preoperative SMI was not associated with the occurrence of postoperative complications (33% vs 35%, *P* = .820), but low preoperative 6MWD was significantly associated with the occurrence of postoperative complications rather than high 6MWD (60% vs 30%, *P* < .001), especially pulmonary complications (23% vs 8%, *P* = .001). In the analysis of long‐term prognosis, low preoperative SMI was associated with poor survival (Hazard ratio [HR] 1.77, *P* = .004). Low preoperative 6MWD was also associated with poor survival (HR 2.55, *P* < .001). Multivariate prognostic analysis showed that pT stage (HR 1.97, *P* = .001), pN stage (HR 3.27, *P* < .001), and 6MWD (HR 1.93, *P* = .008) were independent prognostic factors. In the low 6MWD group, the rate of death from other diseases was significantly higher than the other groups.

**Conclusions:**

It is useful to evaluate 6MWD as a physical performance in addition to SMI when evaluating sarcopenia from the perspective of predicting postoperative complications and long‐term prognosis in patients with esophageal cancer undergoing esophagectomy.

## Graphical abstract

We investigated the usefulness of evaluating physical activity in addition to skeletal muscle mass when evaluating sarcopenia in patients with esophageal cancer. The results showed that preoperative evaluation of physical activity in addition to skeletal muscle mass when evaluating sarcopenia is useful for predicting postoperative complications and long‐term prognosis in patients with esophageal cancer.

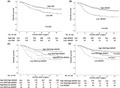

## 1 INTRODUCTION

Esophageal cancer is one of the most malignant cancers. It is the fifth leading cause of death in men and the eighth leading cause of death in women.^1^ Esophagectomy is the standard curative treatment for potentially curable esophageal cancer, but it is highly invasive. Despite recent advances in surgical technique and perioperative management, the frequency of postoperative complications is high and prognosis after resection remains poor.^2^, ^3^ Given this background, predicting the clinical and prognostic outcome of esophageal squamous cell carcinoma is of considerable importance.

Sarcopenia is a disease characterized by progressive loss of skeletal muscle mass and strength.^4^, ^5^ Sarcopenia has been recognized as a functional impairment associated with an increased risk of falls, fractures, and mortality. In recent years, in the evaluation of sarcopenia, various outcomes such as falls, fractures, decreased ability to perform activities of daily living, and death are associated with decreased muscle strength and physical function rather than decreased skeletal muscle mass.^6^, ^7^, ^8^ In esophageal cancer, sarcopenia identified during preoperative evaluation has been reported to be associated with postoperative complications, especially pulmonary complications and anastomotic leakage.^9^, ^10^ Furthermore, it has been reported that sarcopenia in esophageal cancer is associated with poor prognosis.^11^, ^12^ However, when evaluating sarcopenia in patients with esophageal cancer, most studies only used skeletal muscle mass. Few studies that evaluated sarcopenia also considered physical performance. In this study, we investigated whether it is useful to evaluate physical performance in addition to skeletal muscle mass when evaluating sarcopenia in patients with esophageal cancer undergoing esophagectomy, in terms of predicting postoperative complications and long‐term prognosis.

## 2 METHODS

### 2.1 Patients and perioperative treatment

From January 2013 to December 2018, there were 505 consecutive patients with thoracic esophageal cancer who underwent esophagectomy with radical lymph node dissection at the Osaka International Cancer Institute, Japan. Among them, 21 underwent noncurative esophagectomy and 121 patients did not undergo a preoperative assessment of body composition or 6‐minute walk distance (6MWD). After excluding these 142 patients, 363 patients were included in this study, of whom 182 received neoadjuvant chemotherapy and 57 received neoadjuvant chemoradiotherapy.

Our treatment strategy for esophageal cancer has been described in detail previously.^13^, ^14^, ^15^, ^16^, ^17^ Briefly, patients with ≥T2, non‐T4, or node‐positive tumors (Stage ≥1B) received neoadjuvant chemotherapy followed by esophagectomy. Patients who have T4b tumors with suspected invasion of the adjacent trachea, bronchus, or aorta received neoadjuvant chemoradiotherapy. Tumor staging was based on the eighth edition of the Union for International Cancer Control TNM staging system.^18^ The Human Ethics Review Committee of the Osaka International Cancer Institute approved the study protocol (No. 1611259187).

### 2.2 Skeletal muscle mass assessment

Skeletal muscle mass was measured using the body composition method. Within 1 week before surgery, body composition was assessed using multifrequency bioelectrical impedance with eight electrodes (Inbody 720; Biospace).^19^, ^20^ Briefly, various parameters of body composition (body weight, body mass index, total skeletal muscle mass, skeletal muscle mass of the arms and legs, and body fat mass) were measured using the Inbody 720 device. Appendicular skeletal muscle mass was calculated as the sum of the skeletal muscle mass of the four limbs. Skeletal muscle index (SMI) was defined as an appendicular skeletal muscle mass/height^2^. In the present study, low SMI was defined as a SMI of <7.0 kg/m^2^ in men and 5.7 kg/m^2^ in women according to the criteria of the Asian Working Group for Sarcopenia.^21^

### 2.3 Evaluation of physical performance

Functional physical performance was measured with 6MWD within 1 week before surgery prospectively in consecutive patients who underwent esophagectomy for esophageal cancer. The 6‐minute walk test was performed according to the standard procedure described by the American Thoracic Society Committee on Proficiency Standards for Clinical Pulmonary Function Laboratories.^22^ In brief, patients were instructed to walk the predetermined course at their own pace for 6 minutes. Standardized encouragement was given for patients at every minute during the test. At 6 minutes, patients were instructed to stop walking and the distance of the walk measured. 6MWD was measured by physical therapists in the Department of Rehabilitation. Surgeons were blinded to the 6‐minute walk test results.

### 2.4 Postoperative complications

We used the Clavien‐Dindo (CD) classification system to assess complications.^23^ Grade 3 was defined as the need for surgical, endoscopic, or radiologic intervention. Grade 4 was defined as the presence of a life‐threatening complication requiring intensive care unit management. Grade 5 was defined as a cause of death. We identified patients with grade ≥3 complications as having complications.

### 2.5 Statistical analysis

Differences in continuous 6MWD values between groups were evaluated using Student's t‐test. Associations between two categorial parameters were evaluated with the Mann‐Whitney U test, chi‐square test, or Fisher's exact test, as appropriate. Prognostic variables were assessed using the log‐rank test. Overall survival was analyzed using the Kaplan‐Meier method. A multivariate Cox proportional hazards regression model with stepwise comparisons was used to identify independent prognostic markers; variables for which the *P* value in the univariate analysis was <0.05 were included in the multivariate model with a backward stepwise elimination procedure using *P* <.05 as the threshold. All analyses were performed using SPSS for Windows version 27.0.1 (SPSS Japan). Data are expressed as means ±standard deviation. *P* <.05 was considered statistically significant.

## 3 RESULTS

### 3.1 Patient background and distribution of SMI and 6MWD

**FIGURE 1:**
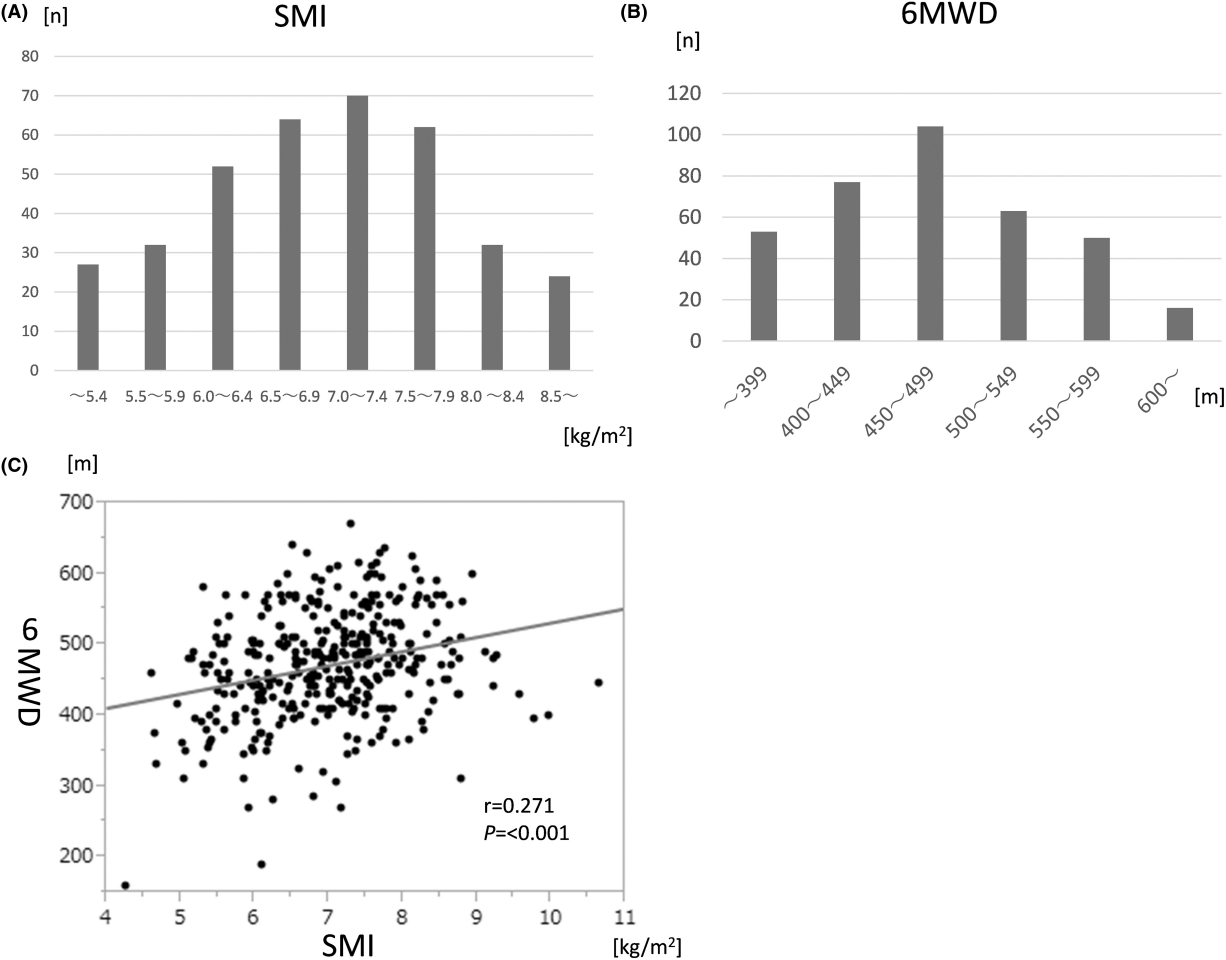
(A) Distribution of SMI, (B) distribution of 6MWD, and (C) the association between SMI and 6MWD. Abbreviations: 6MWD, 6‐minute walk distance; SMI indicates skeletal muscle index

The baseline characteristics of all 363 patients included in this study are summarized in Table S1. There were 244 (67%) patients under the age of 70 and 119 (33%) over the age of 70. There were 290 (80%) males and 73 (20%) females. Preoperative treatment was given to 239 patients (66%): preoperative chemotherapy in 182 patients (50%) and chemoradiotherapy in 57 patients (16%). Figure 1A shows the distribution of SMI measured before esophagectomy. For Inbody 720 SMI measurements, the mean was 7.00 kg/m^2^, median was 7.02 kg/m^2^, standard deviation was 1.01 kg/m^2^, and range was 4.26‐10.66 kg/m^2^. Overall, 224 patients (61.7%) were in the high SMI group and 139 (38.2%) were in the low SMI group, based on the criteria described above. For 6MWD (Figure 1B), the mean was 470.8 m, median was 470 m, standard deviation was 75.7 m, and range was 160‐670 m. 6MWD was 399 m or less in 53 patients (15%), 400‐449 m in 77 patients (21%), 450‐499 m in 104 patients (29%), 500‐549 m in 63 patients (17%), 550‐600 m in 50 patients (14%), and 600 m or more in 16 patients (5%). Figure 1C shows the relationship between 6MWD and SMI. There was a weak correlation between SMI and 6MWD (*r* = 0.271, *P* <.001).

### 3.2 Optimal 6MDW cut‐off value based on survival analysis

The median follow‐up period was 33 months. For all patients, the 3‐year and 5‐year overall survival rates were 71.4% and 66.5%, respectively. We conducted a stepwise analysis that evaluated cut‐off values in 25 m increments, from 375 m to 575 m, to determine the optimal cut‐off for 6MWD for clearly discriminating prognosis for survival, regardless of age or gender. The cut‐off value of 400 m yielded the largest survival difference with the lowest *P* value (hazard ratio [HR], 2.55; *P* <.001; Table 1). There were 53 patients (14.6%) with 6MDW <400 m and a 3‐year survival rate of 51.5%. There were 310 patients (85.4%) with 6MDW ≥400 m and a 3‐year survival rate of 74.9%. Thus, we adopted 400 m as the optimal cut‐off value, which divided patients into two groups from the viewpoint of prognosis. There were 310 patients (85.3%) in the high 6MWD group and 53 patients (14.7%) in the low 6MWD group.

**TABLE 1:** Stepwise analysis of the optimal cut‐off value for 6MWD based on overall survival

6MWD (m)	375	400	425	450	475	500	525	550	575
Number of patients									
Under	33	53	89	130	185	234	278	297	335
Over	330	310	274	233	178	129	85	66	28
3‐year overall survival									
Under	55.0	51.5	64.2	67.9	69.7	68.9	70.5	71.2	70.9
Over	73.0	74.9	73.7	73.4	73.2	76.0	74.1	72.1	77.8
*P* value	0.005	<0.001	0.023	0.064	0.265	0.094	0.333	0.552	0.480
HR	2.15	2.55	1.65	1.44	1.25	1.44	1.27	1.17	1.34

Abbreviations: 6MWD, 6‐minute walk distance; HR, hazard ratio.

### 3.3 Association between SMI or 6MWD and clinicopathological factors

Associations between SMI or 6MWD and clinicopathological factors are shown in Table 2. Compared to the high SMI group, the low SMI group had a higher proportion of older patients (57% vs 26%, *P* <.001), patients with lower height (161.8 vs 166.4 cm, *P* <.001), patients with lower body weight (51.8 vs. 62.3 kg, *P* <.001), patients with lower body mass index (19.8 vs 22.5 kg/m^2^, *P* <.001), patients with advanced pT stage (*P* <.001). Both groups were not significantly different in other factors. On the contrary, compared with the high 6MWD group, the low 6MWD group had a significantly higher proportion of older patients (60% vs 28%, *P* <.001) and males (38% vs 17%, *P* =.001). Compared with the high 6MWD group, the low 6MWD group was shorter (159.4 vs 165.5 cm, *P* <.001) and had lower body weight (54.3 vs 58.9 kg, *P* =.003), a lower proportion of former smokers (72% vs 87%, *P* =.006), a lower prevalence of diabetes mellitus (2% vs 12%, *P* =.027), and a higher proportion of patients with advanced pN stage (*P* =.040). Both groups were not significantly different in other factors.

**TABLE 2:** Patient characteristics by SMI or 6MWD group

Characteristic	Low SMI (n = 139)	High SMI (n = 224)	*P* value	Low 6MWD (n = 53)	High 6MWD (n = 310)	*P* value
Age (years)						
<70	60 (43%)	165 (74%)	<.001	21 (40%)	223 (72%)	<.001
≥70	79 (57%)	59 (26%)		32 (60%)	87 (28%)	
Sex						
Male	105 (75%)	185 (83%)	.108	33 (62%)	257 (83%)	.001
Female	34 (24%)	39 (17%)		20 (38%)	53 (17%)	
Height (cm)	161.8 ± 8.2	166.4 ± 7.4	<.001	159.4 ± 9.2	165.5 ± 7.5	<.001
Body weight (kg)	51.8 ± 7.9	62.3 ± 10.2	<.001	54.3 ± 12.2	58.9 ± 10.2	.003
Body mass index (kg/m^2^)	19.8 ± 2.5	22.5 ± 3.0	<.001	21.3 ± 4.0	21.4 ± 2.9	.801
Smoking						
Yes	120 (87%)	188 (84%)	.453	38 (72%)	280 (87%)	.006
No	19 (13%)	36 (16%)		15 (28%)	39 (13%)	
Alcohol consumption						
Yes	122 (88%)	198 (88%)	.869	43 (81%)	277 (89%)	.106
No	17 (12%)	26 (12%)		10 (19%)	33 (11%)	
Comorbidity						
Heart disease	11 (8%)	26 (12%)	.289	8 (15%)	30 (10%)	.299
Hypertension	55 (40%)	90 (40%)	1.000	25 (47%)	120 (39%)	.288
Lung disease	17 (12%)	6 (3%)	<.001	5 (9%)	18 (6%)	.355
Diabetes mellitus	12 (9%)	25 (11%)	.480	1 (2%)	36 (12%)	.027
Brain disease	7 (5%)	10 (4%)	.803	4 (8%)	14 (5%)	.314
Location						
Upper	34 (24%)	43 (19%)	.127	10 (19%)	66 (21%)	.218
Middle	75 (54%)	112 (50%)		22 (62%)	55 (50%)	
Lower	30 (22%)	69 (31%)		10 (19%)	89 (29%)	
SMI						
High	–	–		21 (40%)	203 (65%)	<.001
Low	–	–		32 (60%)	107 (35%)	
Preoperative treatment						
None	43 (31%)	81 (36%)	.375	12 (23%)	113 (36%)	.119
Neoadjuvant chemotherapy	70 (50%)	112 (50%)		30 (57%)	153 (49%)	
Neoadjuvant chemoradiotherapy	26 (19%)	31 (14%)		11 (21%)	44 (14%)	
pT stage						
pT0	12 (9%)	24 (11%)	<.001	4 (8%)	33 (11%)	.089
pT1	44 (32%)	114 (51%)		17 (32%)	141 (45%)	
pT2	15 (11%)	25 (11%)		4 (8%)	36 (12%)	
pT3	64 (46%)	56 (25%)		26 (49%)	93 (30%)	
pT4	4 (3%)	5 (2%)		2 (4%)	7 (2%)	
pN stage						
pN0	65 (47%)	117 (52%)	.667	21 (40%)	162 (52%)	.040
pN1	47 (34%)	70 (31%)		19 (36%)	98 (32%)	
pN2	19 (14%)	23 (10%)		5 (9%)	36 (12%)	
pN3	8 (6%)	14 (6%)		8 (15%)	14 (5%)	
pM stage						
pM0	12 (9%)	15 (7%)	.540	46 (87%)	290 (94%)	.087
pM1	127 (91%)	208 (93%)		7 (13%)	20 (6%)	
Pathological stage						
pStage 0	9 (6%)	17 (8%)	.081	2 (4%)	25 (8%)	.273
pStage 1	35 (25%)	83 (37%)		14 (26%)	194 (34%)	
pStage 2	35 (25%)	57 (25%)		13 (25%)	79 (25%)	
pStage 3	48 (35%)	52 (23%)		17 (32%)	82 (26%)	
pStage 4	12 (9%)	15 (7%)		7 (13%)	20 (6%)	

Abbreviations: 6MWD, 6‐minute walk distance; SMI, skeletal muscle index.

### 3.4 Impact of SMI and 6MWD on postoperative complications

Table 3 shows the comparison of postoperative complications between the two groups classified based on SMI or 6MWD. For SMI, there were no significant differences in postoperative complications and mortality between the two groups, including pneumonia and anastomotic leakage. However, 32 patients in the low 6MWD group (60%) had complications while 93 (30%) patients in the high 6MWD group had complications (*P* <.001). In particular, pneumonia occurred more frequently in the low 6MWD group (23% vs 8%, *P* =.001). The detailed cause of pneumonia according to the 6MWD is shown in Table S2. The re‐intubation and in‐hospital death also occurred more frequently in the low 6MWD group (11% vs 1%, *P* =.001, and 4% vs 0%, *P* =.021, respectively). Risk factors for postoperative complications were analyzed with consideration of various background factors, including SMI and 6MWD. In the multivariate analysis, low 6MWD was the only significant risk factor for postoperative complications (odds ratio 3.56; 95% confidence interval, 1.96‐6.57; *P* <.001; Table S3).

**TABLE 3:** Association between postoperative complications and SMI or 6MWD

Postoperative complication	Low SMI (n = 139)	High SMI (n = 224)	*P* value	Low 6MWD (n = 53)	High 6MWD (n = 310)	*P* value
Any	46 (33%)	79 (35%)	.820	32 (60%)	93 (30%)	<.001
Pneumonia	12 (8%)	26 (12%)	.112	12 (23%)	26 (8%)	.001
Anastomotic leakage	10 (7%)	13 (6%)	.660	7 (13%)	16 (5%)	.059
Recurrent nerve palsy	9 (6%)	15 (7%)	1.000	6 (11%)	18 (6%)	.139
Chylothorax	6 (4%)	7 (3%)	.572	3 (6%)	10 (3%)	.415
Bleeding	0 (0%)	2 (1%)	.526	1 (2%)	1 (0.3%)	.271
Cardiovascular event	8 (6%)	15 (7%)	.767	5 (9%)	18 (6%)	.469
Surgical site infection	8 (6%)	6 (3%)	.165	1 (2%)	13 (4%)	.702
Other	5 (4%)	9 (4%)	.927	3 (6%)	11 (4%)	.566
Re‐intubation	4 (3%)	5 (2%)	.475	5 (11%)	4 (1%)	.001
Re‐operation	3 (2%)	5 (2%)	1.000	3 (6%)	5 (2%)	.096
In hospital death	1 (1%)	1 (0.5%)	1.000	2 (4%)	0 (0%)	.021

Abbreviations: 6MWD, 6‐minute walk distance; SMI, skeletal muscle index.

### 3.5 Impact of SMI and 6MWD on patient survival

**FIGURE 2:**
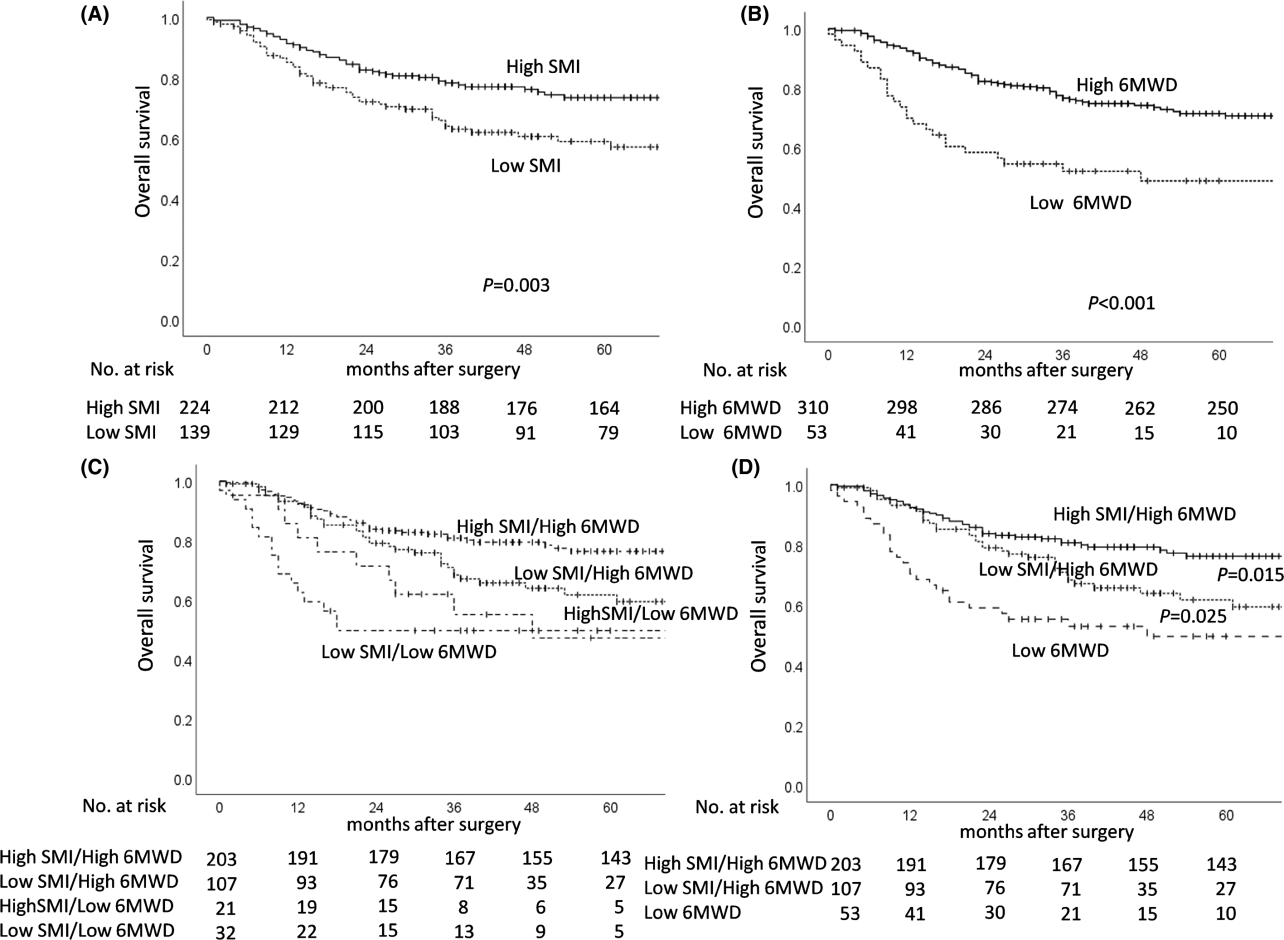
Overall survival by (A) SMI, (B) 6MWD, (C) the combination of SMI and 6MWD in four groups, and (D) the combination of SMI and 6MWD in three groups. Abbreviations: 6MWD, 6‐minute walk distance; SMI indicates skeletal muscle index

The 3‐year overall survival (OS) rates of the high SMI and low SMI groups were 78.4% and 64.0%, respectively. The low SMI group had significantly poorer survival than the high SMI group (*P* =.003) (Figure 2A). The 3‐year overall survival rates of the high 6MWD and low 6MWD groups were 76.6% and 52.0%, respectively. The low 6MWD group had significantly poorer survival than the high 6MWD group (*P* <.001) (Figure 2B).

The univariate analysis of prognosis showed that overall survival was significantly correlated with age (*P* =.040), pT stage (*P* <.001), pN stage (*P* <.001), SMI (*P* =.004), and 6MWD (*P* <.001) (Table 4). The multivariate analysis showed that pT stage (HR, 1.97; *P* =.001), pN stage (HR, 3.27; *P* <.001), and low 6MWD (HR, 1.93; *P* =.008) were independent prognostic factors (Table 4).

**TABLE 4:** Univariate and multivariate prognostic analysis of overall survival

	Univariate analysis			Multivariate analysis		
	Hazard ratio	95% CI	*P* value	Hazard ratio	95% CI	*P* value
Age ≥70 years	1.52	1.02‐2.25	.040	1.07	0.70‐1.60	.749
Male sex	1.31	0.80‐2.28	.291			
pT stage ≥pT3	3.17	2.15‐4.72	<.001	1.97	1.30‐3.00	.001
pN stage ≥pN1	3.70	2.41‐5.85	<.001	3.27	2.08‐5.28	<.001
Low SMI	1.77	1.20‐2.60	.004	1.32	0.87‐1.99	.189
Low 6MWD	2.55	1.62‐3.91	<.001	1.93	1.19‐3.03	.008

Abbreviations: 6MWD, 6‐minute walk distance; SMI, skeletal muscle index.

To investigate the impacts of SMI and 6MWD on survival further, we divided patients into the following four groups: high SMI/high 6MWD, high SMI/low 6MWD, low SMI/high 6MWD, and low SMI/low 6MWD. The number and proportion of patients in these four groups were 203 (56%), 21 (6%), 107 (29%), and 32 (8%), respectively. The high SMI/high 6MWD group had good prognosis, with a 3‐year survival rate of 80.8%, while the low SMI/low 6MWD group had poor prognosis, with a 3‐year survival rate of 49.6%. The 3‐year survival rates of the low SMI/high 6MWD and high SMI/low 6MWD groups were 68.4% and 55.0%, respectively (Figure 2C). Next, we divided patients into three groups, high SMI/high 6MWD group, low SMI/high 6MWD group, and low 6MWD group. The low SMI/high 6MWD group had significantly poorer survival than the high SMI/high 6MWD group (*P* =.015). Furthermore, the low 6MWD group had significantly poorer survival than the low SMI/high 6MWD group (*P* =.025; Figure 2D).

### 3.6 Analysis of the cause of death

Finally, we investigated the association between SMI or 6MWD and the cause of death. Death was due to esophageal cancer in 31 patients (15%) in the high SMI/high 6MWD group, 27 patients (25%) in the low SMM/high 6MWD group, and 16 (30%) patients in the low 6MWD group (*P* =.024). There were eight (4%), seven (7%), and 10 (21%) deaths due to other diseases in the three groups, respectively (*P* =.001; Table 5).

**TABLE 5:** Relationship between prognosis and preoperative SMI and 6MWD

	High SMI/high 6MWD group (n = 203)	Low SMI/high 6MWD group (n = 107)	Low 6MWD group (n = 53)	*P* value
Death due to esophageal cancer	31 (15%)	27 (15%)	16 (30%)	.024
Death due to other diseases	8 (4%)	7 (7%)	10 (21%)	.001

Abbreviations: 6MWD, 6‐minute walk distance; SMI, skeletal muscle index.

## 4 DISCUSSION

In this study, we investigated whether it is useful to evaluate physical performance in addition to SMI when evaluating sarcopenia in patients with esophageal cancer undergoing esophagectomy in terms of predicting postoperative complications and long‐term prognosis. Preoperative SMI was weakly positively correlated with 6MWD. Preoperative SMI was not associated with the occurrence of postoperative complications, but low preoperative 6MWD was significantly associated with the occurrence of postoperative complications, especially pulmonary complications. Furthermore, low preoperative SMI and 6MWD were associated with poor survival, respectively.

This study showed that preoperative SMI was not associated with postoperative complications, whereas low preoperative 6MWD was associated with postoperative complications, especially pneumonia. To date, several studies have reported a relationship between preoperative sarcopenia and postoperative pulmonary complications in patients after esophagectomy for esophageal cancer. Boshier et al reviewed seven studies examining the association between preoperative sarcopenia and postoperative pulmonary complications.^24^ In those seven studies, SMI was evaluated with computed tomography in five studies and the BIA method in two studies. In three studies, low SMI was associated with postoperative pneumonia, and the meta‐analysis showed that SMI was significantly associated with the development of postoperative pneumonia. However, in the present study, the results showed that preoperative SMI was not associated with postoperative complications. On the other hand, regarding 6MWD, only one study by Inoue et al demonstrated an association between preoperative 6MWD and postoperative complications in esophageal cancer.^25^ They reported that low preoperative 6MWD was significantly associated with the development of any complication of CD grade ≥2, consistent with our results. The association between sarcopenia and the occurrence of postoperative complications in esophageal cancer might be slightly related to sarcopenia, which is a decrease in SMI, but might be closely related to sarcopenia due to decreased physical performance. In recent years, according to the sarcopenia evaluation criteria of the Asian Working Group for Sarcopenia 2019 (AWGS 2019) and European Working Group on Sarcopenia in Older People 2 (EWGSOP 2), deterioration of physical function, as measured with 6MWD, reflects the severity of sarcopenia.^21^, ^26^, ^27^ Thus, postoperative complications after surgery for esophageal cancer might be associated with severe sarcopenia. In the evaluation of sarcopenia, predicting the occurrence of postoperative complications by evaluating physical performance in addition to SMI might be effective.

In the present study, the results showed that low preoperative 6MWD was associated with postoperative pneumonia. We also investigated the causes of postoperative pneumoniae. The results showed that the frequency of recurrent nerve palsy was low. Thus, the effect of surgery itself on pneumonia in the low 6MWD group seems to be small. Rather, the main causes of pneumoniae in the low 6MWD group were aspiration immediately after surgery and interstitial pneumonia. These causes might lead to a higher frequency of reintubation. It is presumed that the severe sarcopenia indicated by low 6MWD has decreased swallowing function before surgery, and it is considered that sufficient attention should be paid to pneumonia that occurs immediately after surgery.

The results of this study also suggested that low preoperative SMI was associated with poor prognosis and low preoperative 6MWD was also associated with poor prognosis in esophageal cancer. Both of these factors were independent factors associated with poor prognosis. Deng et al reviewed 11 studies that investigated the association between preoperative sarcopenia and prognosis in patients with esophageal cancer.^28^ Sarcopenia was evaluated with SMI based on computed tomography in 10 reports while sarcopenia was evaluated with the BIA method in one report. The review showed that four studies reported a significant association between sarcopenia and poor prognosis, while the remaining seven studies found that sarcopenia is not associated with poor prognosis. The integrated meta‐analysis of 11 studies concluded that preoperative sarcopenia is associated with poor prognosis. On the basis of these results, preoperative sarcopenia is considered to be associated with poor prognosis in patients with esophageal cancer after esophagectomy. However, it might be difficult to show the relationship between sarcopenia and prognosis by evaluating only muscle mass; controversial results are possible. On the other hand, this is the first report to investigate the relationship between preoperative 6MWD and prognosis in esophageal cancer. We showed that prognosis is poor even with only reduced SMI, but the prognosis was worse in patients with reduced low 6MWD. In other words, by assessing 6MWD in addition to SMI, severe sarcopenia with reduced physical performance could be identified, which could be interpreted as having the worst prognosis among all groups evaluated.

This study also demonstrated that low SMI and low 6MWD are each associated with tumor progression. In the cause of death analysis, the frequency of death from primary disease increased in order from the high SMI/high 6MWD and low SMI/high 6MWD to the low 6MWD group. This finding suggests that the severity of sarcopenia may increase as cancer progresses. Furthermore, compared to the high SMI group/high 6MWD and the low SMI/high 6MWD, low 6MWD group had a frequency of death from other diseases. In other words, the risk of death from other diseases increases as the severity of sarcopenia increases. The severity of sarcopenia might be determined not only by cancer progression but also by factors such as age, nutritional status, and cardiopulmonary function; the combination of these factors might lead to poor prognosis.

In recent years, several researchers have reported the effectiveness of prehabilitation before esophagectomy for esophageal cancer. Minella et al conducted a randomized clinical trial involving 68 patients with esophageal or gastric cancer to examine the effectiveness of prehabilitation consisting of 5 weeks of exercise and nutrition therapy before surgery.^29^ They found that the group receiving preoperative exercise and nutrition therapy had a significant increase in 6MWD. Among 48 patients who underwent esophagectomy for esophageal cancer, Akiyama et al found that 6MWD increased significantly in the patients receiving exercise and nutrition therapy compared with patients who did not receive such therapy.^30^ Thus, to reduce postoperative complications, patients with low preoperative 6MWD might require aggressive preoperative intervention. In the future, it is necessary to investigate whether prehabilitation can decrease sarcopenia, prevent postoperative complications, and improve prognosis.

Several limitations of this study should be acknowledged. First, it was a retrospective cohort study at a single institution. Verification with a multicenter prospective study seems warranted. The second limitation is short follow‐up time. Thus, in the present study, prognosis is analyzed with a 3‐year survival rate. Survival analysis with a longer follow‐up may be needed. The third is that muscle strength was not included in the evaluation of sarcopenia. According to the EWGSOP2 and AWGS2019 sarcopenia evaluation criteria, sarcopenia should be evaluated based on a combination of muscle mass, physical activity, and strength.^25^, ^26^, ^27^ In most study patients, muscle strength could not be measured. Thus, in this study, only muscle mass and physical activity were evaluated. Fourth, 6MWD was used as an indicator of physical performance. In addition to 6MWD, methods for evaluating walking ability, such as gait speed and timed up and go test, and items for evaluating balance ability, such as center of gravity sway test and manual perturbation test, have been reported as evaluation items for physical performance. Which item is the most appropriate index of physical performance before esophageal cancer surgery should be examined in the future.

In conclusion, it is useful to evaluate physical performance in addition to SMI when evaluating sarcopenia from the perspective of predicting postoperative complications and long‐term prognosis in patients with esophageal cancer undergoing esophagectomy.

## DISCLOSURE

Conflict of interest: All the authors have no conflicts of interest to declare. All the authors have no disclosure of funding which should be declared.

Author contributions: Conception and design: K Sugimura and H Miyata; Development of the methodology: K Sugimura, H Miyata, T Kanemura, T Takeoka, M Yamamoto, N Shinnno, T Omori, M Motoori, M Oue, and M Yano; Analysis and interpretation of data: K Sugimura, H Miyata, T Kanemura, T Takeoka, M Yamamoto, N Shinnno, T Omori, M Motoori, M Oue, and M Yano; Writing, review, and revision of the manuscript: K Sugimura and H Miyata.

Ethical Approval: The human ethics review committee of each institution approved the study protocol. This study was performed in accordance with the Declaration of Helsinki.

Informed consent: Subjects provided written informed consent.

## Supporting information

Table S1‐S3
