# Supplementary material for: Impact of preoperative skeletal muscle mass and physical performance on short‐term and long‐term postoperative outcomes in patients with esophageal cancer after esophagectomy
Source: Ann Gastroenterol Surg. 2022 Mar 8;6(5):623–32. doi: 10.1002/ags3.12560 (PMC9444856; doi:10.1002/ags3.12560)
Supplement: Supplementary file 1 — Table S1‐S3 [file AGS3-6-623-s001.docx]

Supplementary Table 1.　Patient background

| Characteristic | |  |  | n=363 |
| --- | --- | --- | --- | --- |
| Age (years) | |  | <70 | 244 (67%) |
|  | |  | ≥70 | 119 (33%) |
| Sex | |  | Male | 290 (80%) |
|  | |  | Female | 73 (20%) |
| Height (m) | | | | 1.65 (1.35–1.86) |
| Body weight (kg) | | | | 57 (35–112) |
| Body mass index (kg/m^2^) | | | | 21.3 (14.1–41.1) |
| Smoking | |  | Yes | 308 (85%) |
|  | |  | No | 55 (15%) |
| Alcohol consumption | |  | Yes | 320 (88%) |
|  | |  | No | 43 (12%) |
| Comorbidity | | | |  |
|  | Heart disease | | | 38 (10%) |
|  | Hypertension | | | 145 (40%) |
|  | Lung disease | | | 23 (6%) |
|  | Diabetes mellitus | | | 37 (10%) |
|  | Brain disease | | | 18 (5%) |
| Tumor location | |  | Upper third | 99 (27%) |
|  | |  | Middle third | 188 (52%) |
|  | |  | Lower third | 76 (21%) |
| Preoperative treatment | | | |  |
|  | None | | | 124 (34%) |
|  | Neoadjuvant chemotherapy | | | 182 (50%) |
|  | Neoadjuvant chemoradiotherapy | | | 57 (16%) |
| pT stage | |  | pT0 | 37 (10%) |
|  | |  | pT1 | 158 (44%) |
|  | |  | pT2 | 40 (11%) |
|  | |  | pT3 | 119 (33%) |
|  | |  | pT4 | 9 (3%) |
| pN stage | |  | pN0 | 183 (50%) |
|  | |  | pN1 | 117 (32%) |
|  | |  | pN2 | 41 (11%) |
|  | |  | pN3 | 22 (6%) |
| pM stage | |  | pM0 | 336 (93%) |
|  | |  | pM1 | 27 (7%) |
| Pathological stage | |  | pStage 0 | 27 (7%) |
|  | |  | pStage 1 | 118 (33%) |
|  | |  | pStage 2 | 92 (25%) |
|  | |  | pStage 3 | 99 (27%) |
|  | |  | pStage 4 | 27 (7%) |

Supplementary Table 2　 The cause of postoperative pneumoniae according to the 6MWD classification

|  | Patients with pneumoniae  in the low 6MWD group  (n=12) | Patients with pneumoniae  in the high 6MWD group  (n=26) |
| --- | --- | --- |
| Recurrent nerve palsy | 1 (8%) | 3 (12%) |
| Aspiration immediately after surgery | 6 (50%) | 13 (50%) |
| Aspiration after starting meal | 0 | 5 (19%) |
| Interstitial pneumonia | 3 (25%) | 0 |
| others | 2 (17%) | 5 (19%) |

6MWD: 6 minute walk distance

Supplementary Table 3　 Univariate and multivariate analysis of postoperative complications

|  | Univariate analysis | | |  | Multivariate analysis | | |
| --- | --- | --- | --- | --- | --- | --- | --- |
|  | Odds ratio | 95% CI | *P* value |  | Odds ratio | 95% CI | *P* value |
| Age ≥70 years | 1.40 | 0.88–2.19 | 0.156 |  |  |  |  |
| Male sex | 1.09 | 0.64–1.90 | 0.753 |  |  |  |  |
| BMI <20 kg/m^2^ | 1.29 | 0.82–2.06 | 0.271 |  |  |  |  |
| Preoperative treatment | 1.13 | 0.72–1.77 | 0.593 |  |  |  |  |
| pStage ≥3 | 1.14 | 0.72–1.81 | 0.579 |  |  |  |  |
| Low SMI | 0.93 | 0.59-1.44 | 0.736 |  |  |  |  |
| Low 6MWD | 3.56 | 1.96–6.57 | <0.001 |  | 3.56 | 1.96–6.57 | <0.001 |

BMI: body mass index, SMI: skeletal muscle index, 6MWD: 6 minute walk distance
